# Heterogeneous PSMA expression on circulating tumor cells - a potential basis for stratification and monitoring of PSMA-directed therapies in prostate cancer

**DOI:** 10.18632/oncotarget.9004

**Published:** 2016-04-26

**Authors:** Tobias M. Gorges, Sabine Riethdorf, Oliver von Ahsen, Paulina Nastały, Katharina Röck, Marcel Boede, Sven Peine, Andra Kuske, Elke Schmid, Christoph Kneip, Frank König, Marion Rudolph, Klaus Pantel

**Affiliations:** ^1^ Department of Tumor Biology, University Medical Center Hamburg-Eppendorf, Hamburg, Germany; ^2^ BPH-DD-TRG-CIPL-Biomarker Research, Bayer Pharma AG, Berlin, Germany; ^3^ ATURO, Urology Practice, Berlin, Germany; ^4^ Department of Transfusion Medicine, University Medical Center Hamburg-Eppendorf, Hamburg, Germany

**Keywords:** CellSearch^®^, circulating tumor cells, prostate cancer, PSMA

## Abstract

The prostate specific membrane antigen (PSMA) is the only clinically validated marker for therapeutic decisions in prostate cancer (PC). Characterization of circulating tumor cells (CTCs) obtained from the peripheral blood of PC patients might provide an alternative to tissue biopsies called “liquid biopsy”. The aim of this study was to develop a reliable assay for the determination of PSMA on CTCs. PSMA expression was analyzed on tissue samples (cohort one, n = 75) and CTCs from metastatic PC patients (cohort two, n = 29). Specific signals for the expression of PSMA could be seen for different prostate cancer cell line cells (PC3, LaPC4, 22Rv1, and LNCaP) by Western blot, immunohistochemistry (IHC), immunocytochemistry (ICC), and FACS. PSMA expression was found to be significantly increased in patients with higher Gleason grade (p = 0.0011) and metastases in lymph nodes (p = 0.0000085) or bone (p = 0.0020) (cohort one). In cohort two, CTCs were detectable in 20 out of 29 samples (69 %, range from 1 - 1000 cells). Twelve out of 20 CTC-positive patients showed PSMA-positive CTCs (67 %, score 1+ to 3+). We found intra-patient heterogeneity regarding the PSMA status between CTCs and the corresponding primary tumors. The results of our study could help to address the question whether treatment decisions based on CTC PSMA profiling will lead to a measurable benefit in clinical outcome for prostate cancer patients in the near future.

## INTRODUCTION

The type II transmembrane protein PSMA (prostate-specific membrane antigen) is highly specific for prostate-derived epithelial cells and has become a clinically validated diagnostically and therapeutically relevant target [1]. Increased expression of PSMA from benign prostatic hyperplasia to high grade intraepithelial neoplasia and to adenocarcinomas [2] as well as overexpression in all stages and grades of prostate cancer (PC) suggest that PSMA plays a pivotal cancer-driving role [3]. Moreover, strong PSMA expression has been associated to higher tumor stages, Gleason scores, preoperative PSA levels, HER2 expression, and to a higher risk of biochemical recurrence [4]. Therefore, PSMA is an attractive therapeutic target and different PSMA-based therapeutic approaches including antibody conjugates, antibody-based radiotherapy, or PSMA-based immunotherapy have been developed and tested in clinical studies validated recently [5]. Since not all PC patients benefit from these therapies, biomarkers predicting response to PSMA-directed therapy are urgently needed.

Currently, decisions for PSMA-targeting therapies are mainly made on the basis of PSMA expression in primary tumors. However, PSMA expression may change during the course of disease but biopsies of recurrent lesions are infrequently performed. Thus, characterization of CTCs obtained from peripheral blood of PC patients might provide an alternative as so-called “liquid biopsy” [6–10].

Here, we present the first study to establish and validate a blood-based assay for real-time analyses of PSMA expression on CTCs in mPC patients, which might establish a basis for future stratification and monitoring of PSMA-directed therapies.

## RESULTS

### Determination of the PSMA status in different prostate cancer cell lines

**Figure 1:**
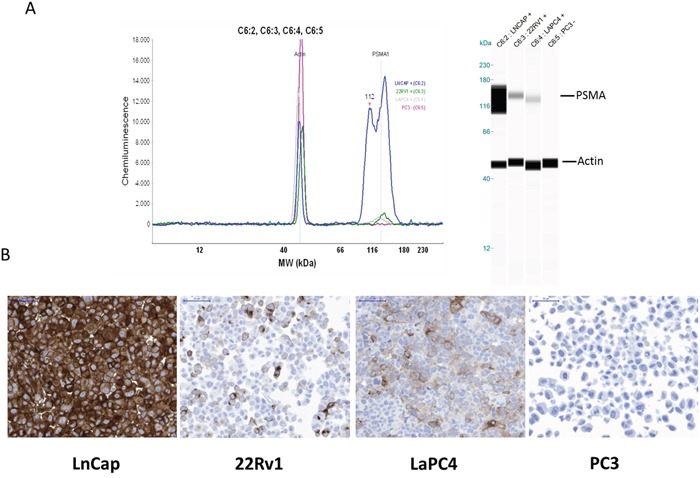
PSMA expression analyses of different prostate cancer cell lines (LNCaP, 22Rv1, LaPC4, and PC-3) by Western blot (A) or immunohistochemistry (B)

PSMA expression of different PC cell line cells was determined by Western blot analysis. Using the DAKO M3620 antibody (clone 3E6), PSMA was clearly detectable in cells of the LNCaP line. Weak PSMA expression was detected in cells of the LaPC4 and 22Rv1 line whereas no PSMA was detected in cells of the PC-3 line. Consistently, similar intensity of PSMA expression was also seen by IHC (Figure 1A and 1B). Immunohistochemical analysis also showed that PSMA expression can be heterogeneous even in cultured cell lines. 22Rv1 and LaPC4 cells demonstrated weak to moderate immunostaining but only in a subpopulation of the cells. Especially in 22Rv1 cells the percentage of PSMA positive cells was low (< 30 %).

### Identification of suitable PSMA antibodies for single cell analysis

First, to implement the CellSearch^®^ system for PSMA determination on CTCs, we had to identify a suitable antibody compatible with the specific fixation and permeabilization used by the CellSearch^®^ system. The antibody used for Western blot and IHC analysis (DAKO, clone 3E6) was not applicable for cell surface immunostaining in flow cytometry or immunocytochemistry under these conditions (data not shown). Thus, additional commercially available FITC-labelled antibodies were tested by flow-cytometry (non-fixed and CellSave®-fixed samples). Using the antibody from EXBIO (clone GCP-05), only weak signals for PSMA could be observed for non-fixed LNCaP and LaPC4 cells, whereas PC-3 cells did not show any signals. However, no PSMA signals could be seen after pre-incubation with the CellSave® fixation reagent (incubation time 24 h) (data not shown). Similar findings were observed when using the antibodies-online antibody (clone 107-1A4) for non-fixed cells. Interestingly, cells of the PSMA-negative cell line (PC-3) showed false-positive staining signals after CellSave®-fixation (clone 107-1A4) (data not shown).

**Figure 2:**
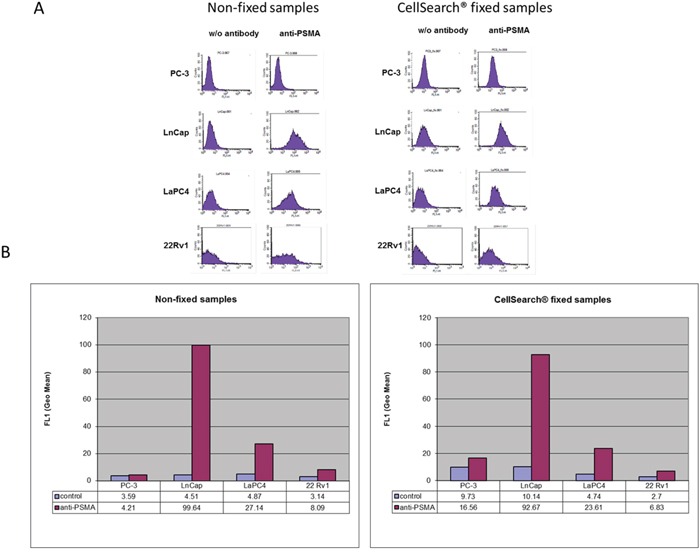
PSMA expression analyses of different prostate cancer cell lines (LNCaP, LaPC4, 22Rv1, and PC-3) by flow cytometry Samples were non-fixed or treated with CellSearch^®^ fixative for 24 hours at room temperature before permeabilization and staining.

PSMA-specific patterns were only obtained (non-fixed and fixed samples) when using the BioLegend antibody (clone LNI-17). No PSMA-specific signals were seen for cells of the PC-3 line whereas moderate to strong signals were observable for cells of the LaPC4 and LNCaP lines (Figure 2A and 2B). Thus, this antibody was chosen for further assay development using the CellSearch^®^ system. A subset of PSMA-positive 22Rv1 cells did not give a clear single peak in the histogram (Figure 2A) but a small increase in positive cells that is in line with the heterogeneous expression as demonstrated by immunohistochemistry (Figure 1B).

### Validation of the antibody for CellSearch^®^ analyses

**Figure 3:**
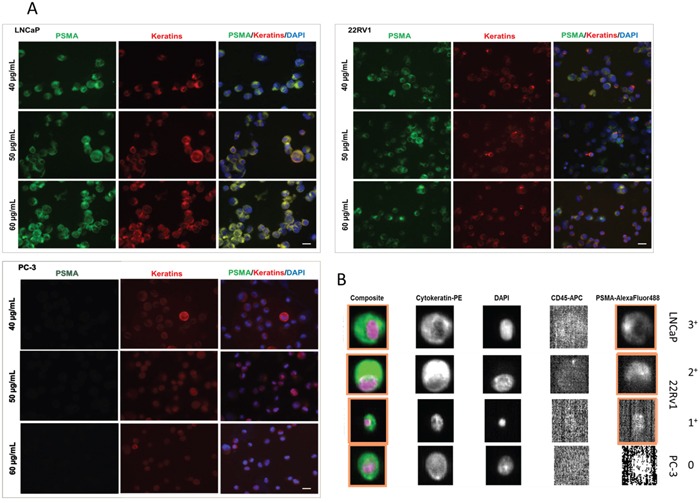
**A.** Immunostaining for PSMA and pan-keratin comparing different prostate cancer cell line cells (LNCap, 22Rv1, and PC-3). DAPI was used for nuclear counterstainings. Different concentrations of the PSMA BioLegend antibody (clone LNI-17) were applied (40 - 60 μg / mL). **B.** Detection of PSMA expression on tumor cells (LNCaP, 22Rv1, and PC-3) spiked into 7.5 mL of healthy donors blood by the CellSearch^®^ system.

Using the BioLegend antibody, intense PSMA-specific signals were present when testing LNCaP cells, whereas 22Rv1 cells only exibited weak to moderate intensity of immunostaining. Applying different concentrations (range between 40 - 60 μg / mL) (Figure 3A), PC-3 cells did not display any PSMA-specific signals. In contrast, LNCaP or 22Rv1 cells presented with moderate to strong intensity of PSMA immunostaining. Due to the heterogeneous staining intensities cells were classified into different scores: “strongly PSMA-positive” CTC (3^+^), “moderately PSMA-positive” (2^+^), and „weakly PSMA-positive” (1^+^). PSMA-negative cells were considered “PSMA-negative” (0). In total, approximately 75 % of the LNCaP cells, 50 % of the 22Rv1, and 30 % of the LaPC4 cell line cells were positive for PSMA.

Next, prostate cancer cells (LNCaP, 22Rv1, and PC-3) were spiked into 7.5 mL blood taken from healthy donors and processed using the CellSearch^®^ system and addition of a FITC-labelled anti-PSMA antibody in the fourth fluorescence channel. After processing, spiked PC-3 cells were PSMA-negative (score 0), whereas LNCaP cells showed intense PSMA immunostaining (75 - 85 % score 3^+^). Cells of the 22Rv1 line exhibited weak to moderate immunofluorescence signals (30 - 40 % score 2^+^ - 1^+^), confirming our previous findings and showing that the sensitivity and specificity of the PSMA antibody is maintained when the samples are processed in the CellSearch^®^ system (Figure 3B). No pan-keratin-positive / PSMA-positive cells could be detected in blood from healthy individuals (n = 8) (data not shown).

Precision, accuracy, and validity of our CellSearch^®^-PSMA assay were tested by two different spike-in strategies. In a first approach, high cell counts were generated by dilution series and spiked into blood from healthy individuals. This approach was also used to study the reliability of our assay over time (one to four days after spiking). In the second approach, cells were spiked-in by manual pipetting of a defined number of single cells. This approach was chosen, since dilution of the cells often leads to differences in cell numbers and biased recovery rates.

**Figure 4:**
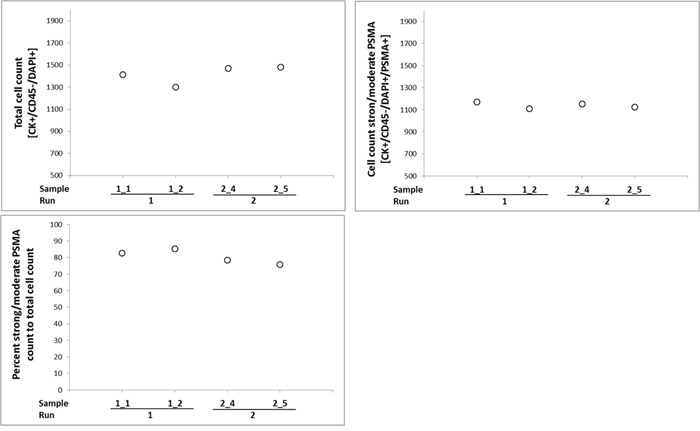
Precision, accuracy, and validity of the CellSearch^®^-PSMA assay Blood from one donor was used, and transferred into CellSave® preservation tubes. Cells of the LNCaP line were spiked into blood by dilution series. Two samples were processed in parallel at day one (samples 1_1 to 1_2) in the same batch / run and two samples (2_4 and 2_5) were processed at day two. Samples 2_4 and 2_5 were stored at room temperature until analysis.

Blood from one donor (40 mL) was used, separated into 5 × 7.5 mL aliquots, and transferred into CellSave® preservation tubes. Approximately 750 cells of the LNCaP line were spiked into blood by dilution series. Three samples were processed in parallel at day one (samples 1_1 to 1_2) in the same batch / run and two samples (2_4 and 2_5) were processed at day two to evaluate if storage of the samples influences the PSMA immunostaining. Samples 2_4 and 2_5 were stored at RT until analysis. Based on the calculated cell numbers used for spike-ins, sometimes recovery rates of greater than 100 % were observed (Figure 4 and supplementary Table 1). These findings are in an acceptable range since spiking by dilution series lacks precision - especially for low cell numbers. Although the intensity of PSMA immunostaining again was heterogeneous among spiked tumor cells, no relevant differences in overall staining patterns were observed when samples were stored over-night. 47 - 50 % of the cells exhibited strong intensity of PSMA immunostaining at day 1 and 50 - 53 % at day two. Similar findings were evident when spiked samples were stored for up to four days at RT, proofing the applicability of the PSMA antibody for long term sample storage (data not shown).

For the second part, 5 (n = 4) and 10 (n = 4) single 22Rv1 cells were spiked into blood from healthy donors and spiked samples were stored in CellSave® tubes for 24 h at RT before processing. All samples (n = 8) were analyzed in one batch / run. Recovery rates from 0 - 120 % could be observed when five cells were spiked into the blood. 0, 25 %, 33 %, and 75 % of the captured cells presented with PSMA-specific immunostaining of weak to moderate intensity. Recovery rates from 60 % - 90 % could be observed when 10 cells were spiked. Within this group, 13 - 25 % of the cells demonstrated PSMA signals of weak to strong intensity. Based on these results, PSMA analysis using CellSearch^®^ was regarded to be of high precision and accuracy for further analysis of clinical samples. However, the observed immunostaining pattern again showed heterogeneity of individual tumor cells even if derived from the same cell line (supplementary Table 2). This heterogeneity was also observed by IHC on tissue samples and is in line with the broad peaks observed in the flow cytometry assay (Figure 1B and 2A).

### Clinical samples

**Figure 5:**
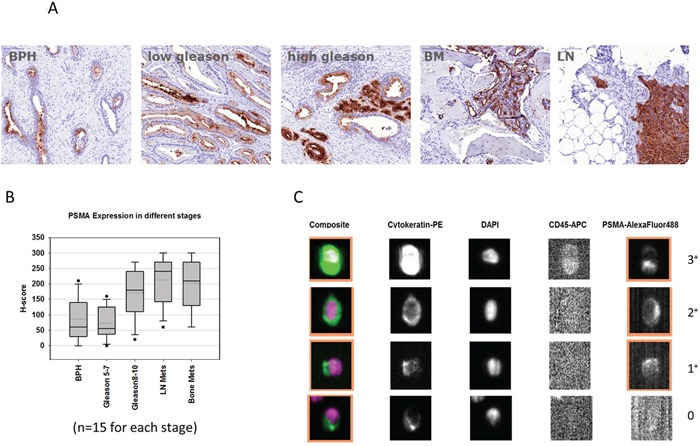
**A and B.** Tissue micro array (TMA) analyses from 75 prostate cancer patients with benign prostate hyperplasia (n = 15), gleason score 5 - 7 (n = 15), gleason score 8 - 10 (n = 15), and patients with bone (BM) or lymph node (LN) metastases (n = 15 each). **C.** Representative images of circulating tumor cells detected by CellSearch^®^ with different signal intensities for PSMA (score 0 - 3+).

To affirm that varying expression levels of PSMA are found at different stages of the disease, clinical samples from 75 PC patients (cohort one; n = 15 per stage; total n = 75) were analyzed by IHC. PSMA expression was significantly increased in patients with higher Gleason grade (p = 0.0011) and metastases found in the lymph nodes (p = 0.0000085) or bone (p = 0.0020) compared to lower Gleason grade tumors (Figure 5A and 5B).

In order to test whether PSMA expression is detectable on CTCs from clinical blood samples (cohort two; n = 29) 7.5 mL blood were analysed by the CellSearch^®^ system. The clinical and pathological characteristics of the patients are summarized in Table 1 (n = 19). Briefly, median age was 68 (range 54 - 90) and the majority of the patients harboured bone (90 %) or lymph node (63 %) metastases. Two patients suffered from additional metastases in the lung or liver. Patients within this cohort were treated with LHRH antagonists (53 %), antiandrogens (63 %), docetaxel-based chemotherapy (21 %) and / or radium-223 (5 %). Laboratory data revealed median values for: PSA 31.13 ng / mL (range 0.15 - 854.46), AP 105.5 U / L (range 37 - 1460), LDH 203.5 U / L (range 103 - 618), and haemoglobin 12.4 g / dL (range 9.1 - 15).

**Table 1:** Patient characteristics

	Number (%) or median (range)	
Age (years)	68 (54-90)	
T Stage		
T1	1 (5 %)	
T2	4 (21 %)	
T3	12 (63 %)	
T4	2 (11 %)	
N Stage		
Nx	6 (32 %)	
N0	1 (5 %)	
N1	12 (63 %)	
Metastasis Location*		*patients may have more than one location
Lymph node	12 (63 %)	
Bone	17 (90 %)	
Lung	1 (5 %)	
Liver	1 (5 %)	
Biopsy Gleason Sum		
7	5 (26 %)	
8 to 10	14 (74 %)	
Years since diagnosis (years)		
Mean	6 (0 - 12)	
Prior (initial) therapy		
only radical prostatectomy	5 (26 %)	
other therapies	14 (74 %)	
Actual therapy*		*patients may have combination of therapies
LHRH agonist/antagonist	10 (53 %)	
Antiandrogen	12 (63 %)	
Chemotherapy	4 (21 %)	
Radium-223	1 (5 %)	
Laboratory data		
PSA (ng / mL)	31.13 (0.15 - 854.46)	
Alkaline phosphatase (U / L)	105.5 (37 - 1460)	
LDH (U / L)	203.5 (103 - 618)	
Haemoglobin (g / dL)	12.4 (9.1 - 15)	
CTC count		
unfavorable (≥ 5 CTCs / 7.5mL blood)	10 (53 %)	
favorable (< 5 CTCs / 7.5mL blood)	9 (47 %)	

Overall, CTCs were detectable in 20 out of 29 samples (69 %, range from 1 - 1000 cells). Twelve out of 20 CTC-positive patients harboured PSMA-positive CTCs (67 %, score 1^+^ to 3^+^). Representative images of PSMA-negative and PSMA-positive CTCs are displayed in Figure 5C. CTCs with strong intensity of PSMA-specific immunofluorescence were detected in 7/12 cases (58 %), however, there was no case with exclusively strongly PSMA-positive CTCs. Thus, all cases showed heterogeneous PSMA phenotypes of CTC. Patients 6, 21, and 29 displayed high percentages of strongly PSMA-positive CTCs (38 %, 43 % and 29 %, respectively, Table 2). According to previous studies on prognostic relevance of CTCs in metastatic prostate cancer, the CTC results were categorized into favorable (< 5 CTCs / 7.5 mL blood) and unfavorable CTC counts (≥5 CTCs / 7.5 mL blood). Interestingly, all CTCs in the favorable CTC count group were PSMA-negative (P-value = 0.031). The CTC-PSMA status was furthermore compared to that of primary tumor tissues from 13 patients. Although we cannot exclude that we missed some PSMA-positive CTCs in the blood samples with lower “favourable” CTC counts, PSMA expression on CTCs might be linked to the rate at which tumors release CTCs and / or the survival of CTCs in the blood stream.

**Table 2:** Detailed patient characteristics and CTC / PSMA results

												Circulating tumor cells						Primary tumor
												Intensity of PSMA immunostaining					PSMA positive CTCs	PSMA Mean
ID	Age	T	N	M	Initial Gleason	Initial PSA ng/ml	tPSA	AP	LDH	HB	Actual therapy	Total No.	neg.	weak	moderate	strong		
1	66	4	1	1	5+4	99	79.88	72	198	10.8	DXI, nsAA	5	5	0	0	0	0 %	65 %
2	68	3	1	0	3+5	-	1.79	62	209	11.8	DXI	1	1	0	0	0	0 %	70 %
3	54	4	1	1	3+4	85	16.97	93	193	13.3	CC, nsAA	0	0	0	0	0	0 %	-
4	74	3	-	1	5+5	389.9	0.05	37	170	12	other	0	0	0	0	0	0 %	80 %
5	61	3	1	1	4+5	>5000	0.29	88	-	13.2	Abirateron, CC	9	9	0	0	0	0 %	80 %
6	83	1	1	1	4+3	7.94	6.12	114	238	12.9	CC	105	0	45	20	40	100 %	30 %
7	72	3	1	1	4+4	150	77.83	106	223	12.8	nsAA, CC	0	0	0	0	0	0 %	-
8	66	2	x	1	3+4	7.8	7.34	-	-	-	Abirateron	24	0	14	10	0	100 %	30 %
9	66	3	x	1	5+4	167	31.96	-	-	-	CC	46	39	0	0	7	15 %	-
10	72	3	1	1	4+5	58	600.99	199	170	14.1	nsAA, CC	9	5	4	0	0	44 %	65 %
11	60	3	1	1	4+5	372	243.22	124	103	14.2	Abirateron, CC	2	2	0	0	0	0 %	20 %
12	62	2	1	1	4+3	43	52.46	-	-	-	nsAA	0	0	0	0	0	0 %	25 %
13	76	3	-	1	4+5	9.5	31.13	82	179	15	CC	0	0	0	0	0	0 %	55 %
14	65	2	-	1	3+4	63	-	118	225	9.1	Abirateron	2	2	0	0	0	0 %	-
15	76	3	0	1	5+5	8.21	854.46	1460	618	11	DXI	426	400	26	0	0	4.50 %	50 %
16	49	3	1	1	4+5	31	18.92	540	157	13.9	DXI, CC	0	0	0	0	0	0 %	-
17	77	2	1	1	4+5	3.1	15.56	402	-	11.1	Abirateron, CC	24	21	3	0	0	12.50 %	7.50 %
18	90	3	-	1	4+5	433	117.59	105	219	10.1	Abirateron	6	6	0	0	0	0 %	90 %
19	72	3	1	1	4+5	13	0.15	68	220	10.6	Abirateron	158	112	23	3	20	29 %	-
20	58	2	1	1	3+4	-	1043	370	293	-	-	8	3	4	0	1	62.50 %	-
21	59	3	1	1	3+4	-	98.89	230	300	11	CC	150	0	85	0	65	100 %	-
22	-	-	-	-	-	-	-	-	-	-	-	3	3	0	0	0	0 %	-
23	68	4	1	1	4+5	7009	427.3	1033	332	9	DXI, CC	1000	0	940	30	30	100 %	-
24	76	3	1	1	4+5	25	12.27	-	-	-	CC	0	0	0	0	0	0 %	-
25	74	3	x	1	4+5	3	0	121	149	12	CC	1	0	0	0	0	0 %	-
26	58	3	1	1	4+5	70	31.24	99	283	15.1	CC	0	0	0	0	0	0 %	-
27	66	4	1	1	5+4	99	86.13	81	268	9	Other (Xofigo)	89	81	8	0	0	9 %	-
28	71	1	1	1	4+5	>100	50.83	68	165	14.1	DXI	0	0	0	0	0	0 %	-
29	65	4	1	1	4+5	-	10	424	216	14.4	CC, nsAA	14	6	4	0	4	57 %	-

DXI = Docetaxel, nsAA = non-steroidal anti-androgene, CC = chemical castration, - = no data)

## DISCUSSION

The major aim of this study was to establish a protocol for the detection of PSMA expression on CTCs. Overall, PSMA expression with striking intra-patient heterogeneity was detectable on CTCs of 12/20 patients with metastatic prostate cancer (67 %).

Bernacki et al. described PSMA as a highly sensitive and specific biomarker for the detection of metastatic prostate cancer cells in cytological specimens [11]. However, the high frequency of PSMA-negative CTCs in patients with PSMA-positive primary tumors detected in our study might be responsible for a lack of response to PSMA-targeting therapies in subpopulations of metastatic tumor cells. PSMA seems to be an ideal target for radiopharmaceutical or cytotoxic antibody conjugates, since antibodies are internalized rapidly after binding to the cell surface. MLN2704, an immunoconjugate designed to deliver an anti-microtubule agent drug directly to PSMA-expressing cells via the PSMA-targeted monoclonal antibody MLN591, shows cytotoxic anti-prostate cancer activity with mild toxicities, predictable pharmacokinetics, and no measurable immunogenicity [12]. Clinical studies with radiolabeled antibodies (MLN591 or J591, a deimmunized monoclonal antibody directed at an external domain of PSMA), have also demonstrated durable antitumor activity [13], illustrating the potential of PSMA as novel target.

PSMA antibodies such as J591 have also been used to capture CTCs from whole blood using a geometrically-enhanced differential immunocapture (GEDI) microfluidic device [14]. Besides, qRT-PCR targeting the *PSMA* transcript was applied to identify CTCs in patients before and after radical prostatectomy [15]. However, to our best knowledge, this is the first study implementing PSMA staining in the FDA-cleared CellSearch^®^ system that offers the possibility to capture CTCs in a standardized and highly reproducible manner within the clinical context. Characterization of CTCs bears a great potential for identifying patients eligible for targeted therapies and may replace the need for invasive procedures. Therapeutic targets such as the HER2, EGFR, or PD-L1 have been analyzed, combined with genetic analysis in other tumor entities [16, 17] and with genomic analysis of resistance genes [18]. In PC, AR signaling was shown to play a pivotal role in carcinogenesis and in particular in the context of anti-androgen therapies [7, 9, 19, 20].

In the present study, PSMA expression of CTCs and corresponding primary tumors was discordant in some patients with lower prevalence of PSMA expression in CTCs. This may be explained by the strong heterogeneity of PSMA expression of CTCs, dynamic changes in RNA or protein expression during EMT [21, 22] or selection of particular CTC subpopulations under therapy.

Taken together, reliable PSMA profiling of individual CTCs in advanced stage PC patients is now feasible and might be used in future studies to stratify PSMA-targeting therapies [23–27]. Current findings show a high expression of PSMA in bone and lymph node metastases, and therefore suggest selection of PSMA-positive clones during progression of the disease. Thus, PSMA-directed therapies should be suitable to block metastatic disease. Future prospective clinical studies have to be designed to address the question whether treatment decisions based on the PSMA profile of CTCs lead to a measurable benefit in clinical outcome for prostate cancer patients.

## MATERIALS AND METHODS

### Cell culture

Prostate cancer cells (PC-3, LaPC4, and LNCaP) were cultured at 37°C (5 % CO_2_) in RPMI cell line medium (Biochrom AG, Berlin, Germany) supplemented with 10 % fetal calf serum (Biochrom AG, Berlin, Germany), 1 % L-Glutamine (Gibco, Carlsbad, CA, US), and 1 % penicillin / streptomycin solution (Gibco, Carlsbad, CA, US). LaPC4 cells were additionally supplemented with 1 nM R1881 (Sigma #R0908, Deisenhofen, Germany). 22Rv1 cells were cultured in 40 % RPMI medium (Biochrom AG, Berlin, Germany) together with 40 % of DMEM medium (Biochrom AG, Berlin, Germany), supplemented with 20 % fetal calf serum (Biochrom AG, Berlin, Germany), 1 % L-Glutamine (Gibco, Carlsbad, CA, US) and 1 % penicillin/streptomycin solution (Gibco, Carlsbad, CA, US).

### Western blot

Western blot analysis for PSMA expression was done using the monoclonal mouse anti- PSMA Clone 3E6 (Dako M3620) at 1:1000 dilution. Western blot signals were evaluated and quantified using the Licor system by Odyssey.

### Immunohistochemistry

We used antigen retrieval for 17 min at 100°C at pH 9 (Target retrieval solution Dako S2367) followed by Protein Block Serum-Free (Dako X0909) for 15 min at RT. The antibody Clone 3E6 (Dako M3620) was added (1:1000 in Antibody-Diluent (Dako S2022)) for 2 h at RT and the EnVision System-HRP (DAB) was used according to the instructions of the manufacturer. Stained slides were scanned at Mirax MIDI slide scanner (3D Histotech).

### Flow cytometry

PSMA expression levels were analyzed by flow cytometry on a FACSCalibur flow cytometer. Briefly, 2 × 10^5^ CellSave-fixed or non-fixed tumor cells (PC-3, LaPC4, 22Rv1, or LNCaP) were used for the analysis. Samples were either non-fixed or treated with CellSearch^®^ fixative for 24 hours at room temperature, washed three times with PBS containing 1 % BSA, then permeabilized by CellSearch permeabilization reagent for 15 min and washed three times with PBS containing 1 % BSA. Cells were then stained using 10 μg/ml in PBS containing 1 % BSA of the FITC-labelled PSMA antibodies (EXBIO #A4-539-C100, clone GCP-05; antibodies-online #ABIN492597, clone 107-1A4, and BioLegend #342506, clone LNI-17) for 30 min at 4°C. After three times washing with PBS containing 1 % BSA, cells were resuspended in PBS and tested by flow cytometry. Subcellular fragments were gated out by forward and side scatter.

### Cytospin preparation

Tumor cell line cells (1 × 10^5^ PC-3, 22Rv1, or LNCaP) were cytospun (Hettich Rotofix 32A) for 3 min at 1200 rpm. Slides were air-dried overnight and fixed for 5 min with 100 μl of the CellSearch^®^ Cell Fixative reagent. Next, slides were washed twice using the CellSearch^®^ dilution solution. Each slide was incubated with a mixture of 30 μl CellSearch^®^ staining reagent (containing fluorescently labelled antibodies against pankeratins and CD45), 10 μl CellSearch^®^ permeabilization reagent, and 30 - 40 μl CellSearch^®^ dilution solution as well as 20 - 30 μl of the anti-PSMA antibody (BioLegend, USA) depending on the final PSMA antibody concentration (40 - 60 μg / mL). Afterwards, slides were washed again, sealed with 4′, 6-diamidino-2-phenylindole (DAPI) Vectashield (Vector Laboratories, USA), and covered with cover-slides for microscopic evaluation.

### CellSearch^®^

The CellSearch^®^ system (Janssen Diagnostics) has been described elsewhere [28]. In this study, CTCs were further characterized for PSMA expression by adding the FITC-labelled BioLegend antibody at a concentration of 50 μg / mL. The criteria for an event to be defined as CTC included: a round to oval morphology, a visible nucleus (DAPI-positive), a positive staining pattern for an epithelial specific cell (pankeratin-positive, PSMA-positive/−negative, and CD45-negative).

### Clinical samples

Cohort one (n = 75): A commercially available tissue micro array (TMA) was purchased from ProVitro (Berlin, Germany) with 15 cores each of the different disease states: benign prostate hyperplasia (BPH), prostate cancer of Gleason Grade 5 - 7, prostate cancer of Gleason Grade 8 - 10 as well as 15 cores each of lymph node metastases and bone metastases derived from prostate cancer patients.

Cohort two: Between November 2014 and December 2015, blood samples from 29 patients with clinically proven mPC were enrolled into this study. The study was carried out in accordance with the World Medical Association Declaration of Helsinki and the guidelines for experimentation with humans by the Chambers of Physicians of the State of Hamburg (“Hamburger Ärztekammer”). The experimental protocol was approved (Approval No. PVN-3779) by the Ethics Committee of the Chambers of Physicians of the State of Hamburg (“Hamburger Ärztekammer”). Clinical and pathological data was collected retrospectively from 19 patients. In this study, blood and matched primary tumor samples from 13 patients were also analysed in parallel for comparative analyses. Peripheral blood from healthy donors was recruited by the Institute of Transfusion Medicine (UKE, Germany). Donors gave general written informed consent to the use of their blood samples in scientific studies.

## SUPPLEMENTARY MATERIAL TABLES


