# Supplementary material for: Heterogeneous PSMA expression on circulating tumor cells - a potential basis for stratification and monitoring of PSMA-directed therapies in prostate cancer
Source: Oncotarget. 2016 Apr 26;7(23):34930–41. doi: 10.18632/oncotarget.9004 (PMC5085200; doi:10.18632/oncotarget.9004)
Supplement: Supplementary file 1 [file oncotarget-07-34930-s001.pdf]

SUPPLEMENTARY TABLES

Supplementary Table S1: Assay repeatability, reproducibility, and accuracy by dilution series over time

Sample	LnCaP		PSMA expression									
			strong		moderate		weak		absent		not evaluable	
	Cells spiked	Cell count (recovery)	Cells	%	Cells	%	Cells	%	Cells	%	Cells	%
Sample 1_1	750	1412	713	50.5	455	32.2	58	4.1	134	9.5	52	3.7
Sample 1_2	750	1301	610	46.9	498	38.3	71	5.5	37	2.8	85	6.5
Sample 2_4	750	1470	736	50.1	417	28.4	70	4.8	40	2.7	217	14.8
Sample 2_5	750	1482	793	53.5	331	22.3	79	5.3	37	2.5	242	16.3
Mean intra-run 1		1356.5	661.5	48.7	476.5	35.25	64.5	4.8	85.5	6.15	68.5	5.1
SD intra-run 1		78.5	72.8	2.5	30.4	4.3	9.2	1.0	68.6	4.7	23.3	2.0
% CV intra-run 1		5.8	11.0	5.2	6.4	12.2	14.3	20.6	80.2	77.0	34.1	38.8
Mean inter-run 2		1416.3	713.0	50.3	425.3	30.3	69.5	4.9	62.0	4.4	149.0	10.3
SD inter-run 2		82.7	76.5	2.7	71.0	6.7	8.7	0.6	48.0	3.4	94.5	6.2
% CV inter-run 2		5.8	10.7	5.4	16.7	22.2	12.5	12.7	77.5	78.1	63.4	59.8

Results for assay repeatability (intra-run) and reproducibility (inter-run / day) as well as accuracy (recovery) in spiking experiments by CellSearch®

Supplementary Table S2: Reproducibility and accuracy after manual spiking

	Recovery			PSMA expression				
Sample	22Rv1	Cells recovered	%	PSMA positive	% of PSMA positive	weak	moderate	strong
1	5	6	120	2	33	2	0	0
2	5	0	0	0	0	0	0	0
3	5	4	80	1	25	1	0	0
4	5	4	80	3	75	1	2	0
5	10	9	90	2	22	2	0	0
6	10	6	60	1	17	0	0	1
7	10	8	80	2	25	0	2	0
8	10	8	80	1	13	0	1	0

Reproducibility and accuracy of the PSMA-detection assay
